# COVID-19 and its effect on emergency presentations to a tertiary hospital with self-harm in Ireland

**DOI:** 10.1017/ipm.2020.116

**Published:** 2020-09-30

**Authors:** A. McIntyre, K. Tong, E. McMahon, A. M. Doherty

**Affiliations:** 1 Department of Psychiatry, University Hospital Galway, Galway, Ireland; 2 University College Dublin, Dublin, Ireland; 3 Mater Misericordiae University Hospital Dublin, Ireland

**Keywords:** Self-harm, self-injurious behaviour, suicide, attempted, COVID-19, psychiatry

## Abstract

**Objectives:**

This study aimed to assess the impact of COVID-19 on presentations to an acute hospital with self-harm.

**Methods:**

All presentations to University Hospital Galway with self-harm were assessed during the peak period of the coronavirus crisis in Ireland, over the 3 months from 1 March to 31 May 2020. These data were compared with presentations in the same months in the 3 years preceding (2017–2019). Data were obtained from the anonymised service database.

**Results:**

This study found that in 2020, the rate of presentation with self-harm dropped by 35% from March to April and rose by 104% from April to May, peaking from mid-May. When trends over a 4-year period were examined, there was a significantly higher lethality of attempt (*p* < 0.001), and significant differences in diagnosis (*p* = 0.031) in 2020 in comparison with the three previous years. The increased lethality of presentations remained significant after age and gender were controlled for (*p* = 0.036). There were also significant differences in the underlying psychiatric diagnoses (*p* = 0.018), notably with a significant increase in substance misuse disorders presenting during the 2020 study period.

**Conclusions:**

COVID-19 showed a reduction in self-harm presentations initially, followed by a sharp increase in May 2020. If a period of economic instability follows as predicted, it is likely that this will further impact the mental health of the population, along with rates of self-harm and suicidal behaviours. There is a need for research into the longer-term effect of COVID-19 and lockdown restrictions, especially with respect to self-harm.

## Introduction

In December 2019, the World Health Organisation (WHO) became aware of a novel coronavirus associated with atypical cases of pneumonia: severe acute respiratory syndrome coronavirus 2 (SARS-CoV-2), in Wuhan, China. This disease, COVID-19 has since then spread internationally and was declared a pandemic by the WHO on 11 March 2020. By 12 June 2020, there were 7 633 217 cases worldwide and 424 495 deaths. COVID-19 is a highly infectious disease with direct neuropsychiatric effects, which are becoming increasingly recognised, along with the effects of the host immunologic response (Rogers *et al.* 2020). There are also however, secondary effects on mental health: these are the indirect effects of the virus on the mental health of the public through its broader societal impact, most notably in the quarantine and physical distancing requirements.

Mental health services have also been required to reconfigure services in order to minimise the spread of coronavirus. People with severe mental illness (SMI) and those with personality disorders are known to have higher rates of physical health co-morbidity than the general population (Prince *et al.* 2007; Fok *et al.* 2012; Chesney *et al.* 2014; Walker *et al.* 2015). As a result of their increased rates of medical co-morbidity, they might be considered an at-risk group for poorer outcomes, should they contract COVID-19 (Kozloff *et al.* 2020). Furthermore, symptoms in SMI including positive symptoms, cognitive impairment and poor insight may impair the ability of people with SMI to follow public health advice, thus increasing their risk of infection (Maguire *et al.* 2019). Poverty, homelessness, substance misuse and living in residential settings further increase the risk of poorer outcomes among people with SMI, in the case of exposure to COVID-19 (Thornicroft *et al.* 2009; Green *et al.* 2018).

As a direct result of the need to reduce transmission of COVID-19 by implementing social (or physical) distancing, many patients attending mental health services have had reduced access to their usual face-to-face mental health supports. Mitigation strategies have necessitated the closure of day hospitals, day centres, treatment groups such as dialectical behavioural therapy and routine face-to-face contact with members of the community mental health team. In many cases, the usual face-to-face contact has been replaced with telephone or video contact. In China, similar limitations placed on mental health services by COVID-19 were associated with relapse of pre-existing mental illness (Yang *et al.* 2020).

Internationally, concerns have been raised regarding the potential impact of social (or physical) distancing on the mental health of populations both with and without pre-existing mental illnesses (Galea *et al.* 2020; Venkatesh & Edirappuli, 2020). Specifically, factors including social (or physical) distancing, reduced access to social supports, physical illness and barriers to mental health treatments were together predicted to reduce the resilience of the population overall and potentially increase the risk of suicide (Reger *et al.* 2020). In addition, it has been posited that increased alcohol consumption, domestic violence and economic sequelae of the COVID-19-related restrictions may contribute to heightened risk of suicidal behaviours (Gunnell *et al.* 2020).

The SARS outbreak in 2003 was followed by a peak in suicides among older adults in Hong Kong (Chan *et al.* 2006). It was associated with increased rates of stress, distress, anxiety disorders including post-traumatic stress disorder, and depressive illnesses (Lee *et al.* 2007; Mak *et al.* 2009). Given the similarities with COVID-19, a risk of similar population increases in depressed mood, anxiety and suicidal thoughts was predicted in response to the 2020 COVID-19 pandemic (Torales *et al.* 2020). The reductions in normal social interactions and activities and resulting social isolation may potentiate these difficulties, given that in addition to being important for wellbeing, these are also protective against suicide (Zadravec Šedivy *et al.* 2017).

There were real concerns about the practical management of mental health crises, due to the combination of reduced face-to-face supports and increased social isolation (Yao *et al.* 2020). In addition, the restrictions in visiting hospitals and changes to emergency pathways might deter people from attending hospital when needed in a crisis. Locally, in Galway, an alternative pathway for the management of mental health crises was developed to allow for the safe assessment of people with mental disorders presenting acutely, away from the COVID-19 pathway.

Self-harm is an important predictors of suicide and as such represents an important opportunity to intervene (Carroll *et al.* 2014; Bostwick *et al.* 2016). There is little published regarding self-harm in relation to COVID-19: internationally, there have been three cases attributed to COVID-19 in case reports from India and Germany (Sahoo *et al.* 2020; Weise *et al.* 2020).

This paper aims to assess the impact of COVID-19 and the associated social change on rates of self-harm at a tertiary university hospital in Ireland, by comparing the levels of self-harm during the periods of the pandemic with the same months and weeks in three previous years: 2017, 2018 and 2019. It aims to examine the methods and severity of self-harm and suicidal behaviours, and the associated diagnoses and treatments.

## Method

The Liaison Psychiatry Team (LPT) at University Hospital Galway (UHG) maintains a database of all patients who are referred for assessment by the service, both within the working hours of the team and out of hours. UHG is a tertiary university hospital on the west coast, with 850 beds over 2 sites. It is the only Model 4 hospital in the Saolta Group and as such receives complex specialty referrals from the other group hospitals, some of which are over 200 km distant. These include urgent referrals to plastic surgery in the case of severe self-lacerations requiring surgical intervention. The LPT is a team of psychiatrists and mental health nurses which provides psychiatric consultations and treatments to patients admitted to medical and surgical wards and those attending the emergency department (ED). In 2020 during the study period, it was staffed at under 30% of national and international standards (DoH, 2006; PLAN, 2014).

The service database includes patients referred from the ED, the medical and surgical wards, and the critical care unit. It includes basic demographic and clinical information, including age, gender, reason for referral, diagnosis, and treatments including whether or not the person is admitted to the acute adult mental health inpatient unit. Information was extracted on patients referred to the service with self-harm in the 3 months at the height of the pandemic: 1 March to 31 May 2020. These were compared to the referrals received from 1 March to 31 May in the three preceding years: 2017, 2018 and 2019.

Self-harm was defined as per the WHO as ‘*an act with non-fatal outcome, in which an individual deliberately initiates a non-habitual behaviour that, without intervention from others, will cause self-harm, or deliberately ingests a substance in excess of the prescribed or generally recognised therapeutic dosage, and which is aimed at realising changes which the subject desired via the actual or expected physical consequences’* (Platt *et al.* 1992).

We used source of referral as a surrogate for severity of self-harm: we categorised those requiring admission to critical care as the most severe (these patients would likely have died without treatment); those requiring medical or surgical admission were moderately severe as the toxicity or injuries inflicted required inpatient treatments; and those who could be discharged home from the ED or equivalent were the least severe as they did not require inpatient medical treatment. This measure gives an approximation of lethality, although it was not possible to capture the degree of suicidal intent from the database.

For the purpose of this study, the anonymised data were exported to an SPSS file. Given the changing nature of the restrictions over time, data were broken down by month and week for 2020. Chi-square was used to compare the categorical variables, and independent sample *t*-test was used to compare the scale variables. We used a logistic regression model to control for age and gender.

Ethical approval was obtained from the Clinical Research Ethics Committee of the Saolta Hospital Group (no. 2386). As all data were anonymised, written consent was not required.

## Results

**Fig. 1.**
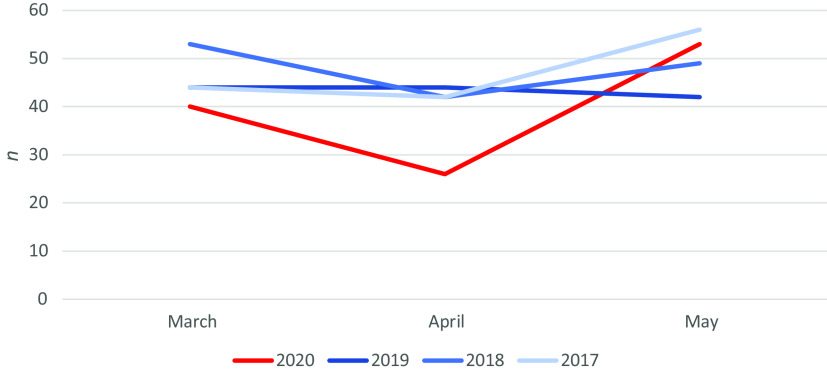
Self-harm presentations by month March–May over 4 years 2017–2020.

**Fig. 2.**
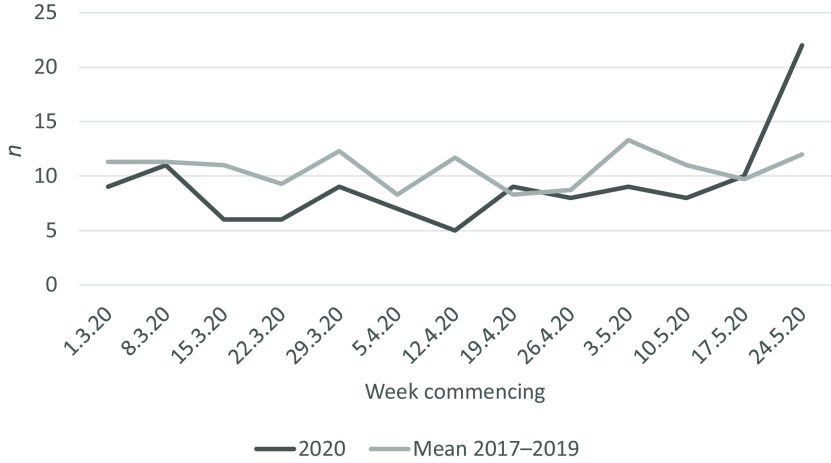
Self-harm presentations by week March–May compared with mean 2017–2019.

From 1 March to 31 May 2020, there were 576 referrals to psychiatry compared with 760 in the same months in 2019: an overall reduction of 31.9%. From 1 March to 31 May 2020, there were 119 referrals with self-harm compared with 130 in the same months in 2019: a reduction of 8.5%: the decrease in presentations with self-harm was less than the decrease in attendances for other psychiatric indications. In 2020, the rate of presentation with self-harm dropped by 35% from March to April but rose by 104% from April to May (Fig. 1). The degree of variability week to week was considerable, but throughout the first 10 weeks of COVID-19 the rates remained constant (range 5–11/week), with a precipitous increase from mid-May (Fig. 2).

Table 1 describes the characteristics of those patients who were referred for a psychiatric assessment with self-harm. There were no significant differences in the age or gender of patients referred in 2020 compared with previous years.

**Table 1.** Demographic and clinical characteristics of patients presenting to the emergency department with self-harm in the 3 months of the 2020 COVID-19 crisis, compared with those presenting in the preceding 3 years

	2020 (*n* = 119)	2017–2019 (*n* = 406)	*p*-Value
Gender			
Female, *n* (%)	68 (57.1)	254 (61.1)	0.442^a^
Male, *n* (%)	51 (42.9)	162 (38.9)	
Age, mean (SD)	34.1 (15.8)	31.6 (15.1)	0.106^b^
Age			
<18 years, *n* (%)	18 (15.1)	73 (17.5)	0.668^a^
18–64 years, *n* (%)	94 (79)	325 (78.1)	
>65 years, *n* (%)	7 (5.9)	18 (4.3)	
Source of referral			
Emergency department, *n* (%)	84 (70.6)	355 (85.3)	<0.001^a^
Medical/surgical wards, *n* (%)	29 (24.4)	37 (8.9)	
Critical care, *n* (%)	6 (5)	24 (5.8)	
Method of self-harm			
Poisoning, *n* (%)	85 (71.4)	188 (61.6)	0.069^a^
Cutting, *n* (%)	20 (16.8)	71 (23.3)	
Drowning, *n* (%)	1 (0.8)	17 (5.6)	
Hanging, *n* (%)	5 (4.2)	16 (5.2)	
Other, *n* (%)	8 (6.7)	13 (4.3)	
Diagnosis			
Depressive episode, *n* (%)	22 (18.6)	91 (22.8)	0.031^a^
Adjustment disorder, *n* (%)	20 (16.9)	54 (13.5)	
Personality disorder, *n* (%)	18 (15.3)	79 (19.8)	
Substance misuse, *n* (%)	36 (30.5)	87 (21.8)	
Anxiety disorders, *n* (%)	8 (6.8)	19 (4.8)	
SMI (psychosis and BPAD), *n* (%)	10 (8.5)	22 (5.5)	
Other	4 (3.4)	47 (11.8)	
Treatment plan			
Admission, *n* (%)	18 (15.1)	73 (17.5)	0.46^a^
CMHT, *n* (%)	90 (75.6)	298 (71.6)	
Addictions, *n* (%)	3 (2.5)	23 (5.5)	
Primary care, *n* (%)	8 (6.7)	22 (5.3)	

SMI, severe mental illness; BPAD, bipolar affective disorder; CMHT, community mental health team.

a Chi-square.

b Independent sample *t*-test.

There were significant differences in source of referral (i.e. from the ED, inpatient wards or critical care) a proxy for lethality (*p* < 0.001) with a significant increase in numbers of patients requiring medical or surgical admission for management of their self-injury or poisoning; from 8.9% to 24.4% of patients presenting with self-harm (2.7-fold). There were no significant changes in method of self-harm, although there were trend increases in self-poisoning and trend reductions in cutting.

There were significant differences in the underlying diagnoses – in particular, an increase in primary substance misuse presentations: from 21.8% to 30.5% (*p* = 0.031). There was a trend increase in people with a diagnosis of SMI presenting with self-harm: from 5.5% to 8.5%. There was a trend increase in the proportion of people with adjustment disorders and with anxiety disorders, and a decrease in those with depressive episodes and with personality disorders.

There were no significant differences in treatment between the stude period and the comparison periods in the previous 3 years. Rates of psychiatric admissions of people presenting with self-harm dropped from 17.5% to 15.1%, but this change was not statistically significant.

On logistic regression, after controlling for age and gender, the differences in severity or medical lethality of self-harm remained significant between 2020 and previous years (OR 1.47; *p* = 0.036; 95% CI 1.023–20.72), that is, there was a significant increase in patients requiring medical admission for treatment (Table 2). The differences in diagnosis also remained significant (OR 1.38; *p* = 0.018; 95% CI 1.236–17.78).

**Table 2.** Logistic regression with year (2020 *v.* earlier years) as the dependent variable

	Odds ratio	*p*-Value	95% CI
Gender	0.893	0.596	0.587–1.358
Age	1.007	0.332	0.983–1.020
Source of referral/severity^a^ of self-harm	1.46	0.037	1.023–2.072

a Referral source was used as a surrogate measure for severity, with referrals from critical care most severe, patients who required medical or surgical admission considered moderately severe, and those not requiring inpatient treatment considered least severe.

## Discussion

This study shows that there was a small overall reduction of 8.5% in the numbers of patients presenting with self-harm from 1 March to 31 May 2020, compared with the same period in 2019. When trends over a 4-year period were examined, there were no significant changes in the patterns of age or gender in those presenting with self-harm in 2020 compared with previous years. There was a significant increase in the lethality of presentations reported and this remained significant after age and gender were controlled for. There were significant differences in the underlying psychiatric diagnoses, notably a significant increase in substance misuse disorders and SMI.

This study reported low levels of self-harm during the month of April, particularly in the week of 12 April, which was the month and week where Ireland recorded the highest number of COVID-19 cases throughout the period of the outbreak (CovidwatchIrl, 2020; DoH, 2020). It is possible that people who are at risk or may have self-harmed may not have sought medical attention in the hospital setting if they perceived that the self-harm was not severe enough as to require immediate medical attention, due to the severity of the pandemic in the country at that time. Fear among the public about attending hospital during the lockdown dissuaded many with serious or potentially life-threatening conditions from seeking medical care, including those with strokes and acute coronary syndromes (Kristoffersen *et al.* 2020; Mafham *et al.* 2020). During the early stages of the lockdown, it is possible that only those with the most severe self-harm attended hospital. In May, the steep increase in the number of cases of self-harm coincides with the easing of restrictions in the country, with Phase 1 of easing of the lockdown beginning on 18 May (DoH, 2020). It is likely that as people become more relaxed about COVID-19, the public is more willing to present to the hospital for help with non-COVID-19-related conditions, including self-harm. Another reason for the sharp rise could be the aftermath of a national lockdown, with cocooning for the high-risk population.

Previous viral epidemics have been accompanied by peaks in rates of suicide: this was first reported following the 1918 ‘Spanish’ influenza pandemic in the US (Wasserman, 1992), and more recently following the SARS outbreak in Hong Kong (Chan *et al.* 2006). Like in the case of the SARS outbreak in 2003, this study provides early evidence that the COVID-19 outbreak may be associated with increased rates of anxiety disorders and with increased severity of self-harm and suicidal behaviours (Chan *et al.* 2006). There is little published on any relationship which may exist between COVID-19 and self-harm, with three cases attributed to its societal sequelae reported from India and Germany (Sahoo *et al.* 2020; Weise *et al.* 2020). The two cases from India appear to have been of depressive episodes triggered by health anxiety, and the case in Germany related to a delusional disorder: none of the three patients described had any psychiatric history. Similarly, in this study, there were increased rates of patients presenting with self-harm on a background of anxiety disorders and psychotic illnesses. In this study, the association of self-harm with a primary diagnosis of substance misuse disorders is of concern and may reflect an increase in alcohol consumption in the general population.

People with self-harm with more serious injuries or poisoning require a medical or surgical admission, and the most gravely ill with a life-threatening status may require treatment in the critical care unit. This range of presentations may be regarded as a spectrum of lethality, with suicidal ideation at one end and completed suicide at the other (Mohan *et al.* 2020). This conceptualisation of lethality suggests that patients who require critical care interventions may be more similar to those who die by suicide, than those requiring brief review only. It is unclear to what degree suicidal intent is associated with suicidal lethality.

Regarding future trends, the economic fallout of the crisis will be important. There is evidence that economic crises are associated with poorer mental health outcomes and increased rates of suicides with evidence that every 1% increase in unemployment is associated with a 0.79% increase in rates of suicide (Stuckler *et al.* 2009). In March 2020, the International Labour Organization estimated that global employment would decline by between 5.3 and 24.7 million (I.L.O., 2020). Based on these projected figures of unemployment as a result of COVID-19, it is predicted that there would be a rise of between 2135 and 9570 suicides per year (Kawohl & Nordt, 2020), from the base level of nearly 800 000 suicides annually (WHO, 2019a). This figure does not account for episodes of self-harm which is postulated to be increasing. According to WHO, for every suicide, there are over 20 suicide attempts (WHO, 2019b), and existing research has shown that self-harm and suicidal behaviour is a strong risk factor for death by suicide (Carroll *et al.* 2014; Bostwick *et al.* 2016).

The limitations of this study include its retrospective design and its basis on a service database which out-ruled the incorporation of patient-reported measures. The use of a database might also be considered to be a strength, as all patients presenting with self-harm were included, removing participation or selection bias. In the absence of a patient-reported measure of intent or perceived lethality, the degree of medical support required provides a surrogate marker of severity.

This is the first study to examine variations in rates of self-harm associated with the COVID-19 pandemic in 2020, and it provides early evidence that there may be changes in rates of self-harm associated with the crisis.

There is a need for further research into the longer-term effect of the restrictions and changes due to COVID-19 over the months ahead. If a period of economic instability follows as predicted, this may have a further impact on the mental health of the population, and perhaps on rates of self-harm.

## Conclusion

COVID-19 showed a reduction in self-harm presentations initially, followed by a sharp increase in May 2020. If a period of economic instability follows as predicted, it is likely that this will further impact the mental health of the population, along with rates of self-harm. There is a need for research into the longer-term effect of the restrictions and changes due to COVID-19 on mental health, mental illness and suicidality. There is evidence of the need for mitigation measures to ensure psychiatric services are adequately resourced to provide a high standard of care for those who require it.
